# Incidence of different pressure patterns of spinal cerebellar ataxia and analysis of imaging and genetic diagnosis

**DOI:** 10.1515/biol-2022-0762

**Published:** 2023-12-12

**Authors:** Yufen Peng, Qi Tu, Yao Han, Liang Gao, Chenyi Wan

**Affiliations:** Department of Neurology, The First Affiliated Hospital of Nanchang University, Nanchang, Jiangxi, 330006, China

**Keywords:** spinal cerebellar ataxia, heredity, sporadic, genetic diagnosis

## Abstract

Neurologists have a difficult time identifying sporadic cerebellar ataxia. Multiple system atrophy of the cerebellar type (MSA-C), spontaneous late cortical cerebellar atrophy, and prolonged alcohol use are a few possible causes. In a group of people with sporadic cerebellar ataxia that was not MSA-C, an autosomal-dominant spinocerebellar ataxia (SCA) mutation was recently discovered. Chinese single-hospital cohort will be used in this study to genetic screen for SCA-related genes. One hundred forty individuals with CA were monitored over 8 years. Thirty-one individuals had familial CA, 109 patients had sporadic CA, 73 had MSA-C, and 36 had non-MSA-C sporadic CA. In 28 of the 31 non-MSA-C sporadic patients who requested the test, we carried out gene analysis, including SCA1, SCA2, SCA3, SCA6, SCA7, SCA8, SCA12, SCA17, SCA31, and dentatorubro-pallidoluysian atrophy (DRPLA). The control group consisted of family members of the patients. In 57% of the instances with spontaneous CA that were not MSA-C, gene abnormalities were discovered. The most frequent exception among individuals with sporadic CA was SCA6 (36%), followed by monsters in SCA1, 2, 3, 8, and DRPLA. In contrast, 75% of the patients with familial CA had gene abnormalities, the most frequent of which was SCA6 abnormality. The age of 69 vs 59 was higher, and the CAG repeat length was a minor age of 23 vs 25 in the former instances compared to the last one among individuals with SCA6 anomalies that were sporadic as opposed to familial cases. In sporadic CA, autosomal-dominant mutations in SCA genes, notably in SCA6, are common. Although the cause of the increased incidence of SCA6 mutations is unknown, it may be related to a greater age of onset and varied penetrance of SCA6 mutations.

## 1 Introduction

Spinocerebellar ataxias (SCAs) are a distinct autosomal dominantly inherited neurological disease category. Nearly 50 subtypes have now been identified. In advance of this, a systematic study indicated 2.7 incidences of SCA per 100,000 people worldwide [1]. Cerebellar atrophy is a key pathological feature of SCAs, and ataxia gait is often the first symptom, according to clinical and pathological research. Cerebellar symptoms such as stumbling gait, slurred speech, and nystagmus are the usual clinical signs of SCAs. Various non-cerebellar symptoms, including extrapyramidal symptoms, dystonia, retinopathy, peripheral neuropathy, and Parkinsonian syndromes, may also be present in SCAs [2]. Clinical variation exists in many SCA subtypes and within a single subtype. Any reliable biomarker cannot yet determine the disease state of SCA. The most typical sign that SCAs are beginning is abnormal gait. The most often used metrics for judging the clinical gaits of SCA patients are ataxia measures, specifically semi-quantitative scales [3].

Given that over 60% of the world’s population resides on the Asian continent, most SCA patients are either of Asian ancestry or residents of the Asian continent. Despite this, Asian nations provide a disproportionately small amount of scientific evidence on SCA. A few nations account for the bulk of research, whereas many other nations currently need to be represented in the literature [4]. This indicates that for many Asian nations, information about the frequency of SCA, genetic subgroups, and illness symptoms is lacking. It is becoming more crucial than ever for SCA patients to be identified early on and for a specific SCA genotype to be determined since novel treatment options for SCAs are on the horizon, some of which are very selective for certain genotypes [5]. In Asia, especially China, where Friedreich’s ataxia is uncommon, 17.3% of 237 individuals in sporadic cerebellar ataxia had a dominant SCA mutation that causes the condition [6]. These mutations include 23 SCA3, 9 SCA2, 6 SCA1, and 3 SCA6 mutations. Given that SCA6 seems to present later than other forms of SCA, nine it is possible that people with sporadic cerebellar ataxia are more likely to have mutations in the CACNA1A gene, which causes SCA 6 [7]. To support this theory, we genetically tested SCA genes in a group of Chinese patients from a single hospital.

## 2 Analysis of imaging and genetic diagnosis in SCA

Ghorbani et al. [8] included cerebellar ataxia patients sent to the University Medical Center Groningen for SCA genetic tests. The Infinium Global Screening Array has been employed to carry out genome-wide single nucleotide polymorphism genotyping. After processing the data, genotyping data were input into the NxClinical application to conduct Copy number variant (CNV) assessment for each patient and to demonstrate CNVs in 36 genes with assigned SCA symbols. Using data from research that uses PubMed searches in the literature for comparable CNVs and phenotypic characteristics, the clinical significance of discovered CNVs was established. Yang et al. [9] assessed the levels of neurofilament light (NfL) in SCA type 2 (SCA2) patients and confirmed if these levels are related to the severity of the condition. Patients having SCA2 were genetically identified, and participants were drawn from one Chinese medical facility. The single molecule array technique was used to measure NfL levels. Patients having SCA2 were genetically determined, and participants were removed from one Chinese medical facility. The single molecule array technique was used to measure NfL levels.

Arias [10] analyzed the most prevalent autosomal recessive SCA types. A comprehensive evaluation of the clinical phenotype, lab tests, nerve conduction investigations, and a magnetic resonance imaging (MRI) examination may aid in making a preliminary diagnosis that should be verified by identifying the underlying genetic mutation. For prognosis, good genetic counseling, and certain entities’ proper treatment, a positive genetic test result is required. It can only conduct fundamental research or clinical trials with a genetic diagnosis. Giardina et al. [11] described an instance of late-onset oro-facial dyskinesia of an older patient with SCA2 as a genetic diagnosis. Huntington’s disease is often the only genetic condition that causes oro-facial dyskinesia; other states are frequently overlooked or underappreciated. SCA2 is one of the potential reasons for adult-onset orofacial dyskinesia, mainly if there is evidence from the family history of an inherited cerebellar condition. Clinical features like Parkinsonism and motor neuron disease could be relevant indicators for a prompt diagnosis and effective treatment. Agarwal et al. [12] assessed the level of cognitive impairment in people with SCA type 12 (SCA12) diseases. Patients with several SCAs are now known to have cognitive impairment. Patients having SCA12 have not yet been studied for cognitive impairment and conducted cross-sectional research, enrolling 30 SCA12 patients with genetic confirmation and 30 healthy, age-, gender-, and∼education-matched controls. Several established neurocognitive tests were used to evaluate various cognitive areas.

Starting in November 2019, individuals from Huashan Hospital and the CABLE project were recruited one after the other for the research [13]. Patients diagnosed with spinal cord atrophy (SCA) were genetically analyzed, ranked according to the severity of their ataxia, and contrasted with patients diagnosed with multiple system atrophy of the cerebellar type (MSA-C). Healthy older persons were also included in the comparison. Simoa performed tests on all subjects to determine the plasma NfL, GFAP, p-tau, and A levels. To investigate potential indicators associated with SCA, we used a study of covariance findings, a Spearman correlation, and a multivariable regression. van Prooije et al. [14] provided a synopsis of three primary characteristics of SCA in Asian populations. A meticulous examination of epidemiological research was carried out. In general, SCA1, SCA2, SCA3, and SCA6 are the most prevalent subtypes, although the exact incidence of all of these SCA subtypes varies significantly among the nations of Asia. They provided a synopsis of the phenotypic manifestations associated with SCA patients of Asian descent. One such symptom is the frequent co-occurrence of Parkinsonism in specific SCA subtypes.

In conclusion, exploratory survey research was conducted to map SCA-specific skills, resources, and management across various Asian nations. Schmitz‐Hübsch et al. [15] investigated data on the brain’s neurological, cognitive, and imaging properties that were prospectively collected from 33 individuals with the protein kinase C gamma (PRKCG) variation. The use of protein modeling as criteria for categorization in interpretations of uncertain significance (VUS) was recently implemented. In the most significant cohort studied to date, SCA-PRKCG was found to be a cerebellar syndrome that progressed slowly over time and had specific clinical and imaging symptoms indicative of a developmental illness. The cerebellar disease is likely the cause of the reported non-ataxia movement abnormalities and cognitive-affective disturbance.

Chiu et al. [16] identified two spinocerebellar ataxia type 16 (SCAR16) pedigrees from 512 cerebellar ataxia-afflicted Taiwanese households. Regarding their clinical conditions, the three individuals from the two SCAR16 families all had cerebellar ataxia alone or in conjunction with cognitive impairment. The patient’s cerebellar atrophy was seen on the brain MRIs. Rentiya et al. [17] examined the most common SCA subtypes according to their genetic origin, etiology, neurological manifestations, additional presenting symptoms, and diagnostic workup. The quest for a treatment for SCA, which is now incurable, should be one of the other research objectives in this area. Saucier et al. [18] analyzed the typical beginning age was 59.1 years old, with symptoms including dysarthria and ataxia that developed gradually over time. Coulometer malfunction, dysphagia or hypoalgesia, vibratory hypoesthesia, and dysreflexia were typical clinical characteristics. Abnormal movements, extrapyramidal symptoms, and cognitive impairment may sometimes be noted in patients with this condition. Radiological examinations will likely reveal a high incidence of atrophy affecting the cerebellum, which may sometimes be accompanied by atrophy affecting the brainstem and the cortices.

Alshimemeri et al. [19] discussed the prevalence of autosomal dominant (AD) SCA and its distribution and clinical features. It identified significant differences in most frequent SCA among all three investigated provinces. This conclusion shows the diverse character of Canada’s population reflected in its heterogeneity. Ghorbani [20] examined a group of patients who had yet to be solved to search for genetic variants in all genes known to have a role in the development of SCA. They were able to identify the genetic root of 22 individuals’ conditions. The fact that geneticists can identify gene variation without determining its cause is a significant obstacle for diagnostics [21].

## 3 Materials and methods

**Figure 1:**
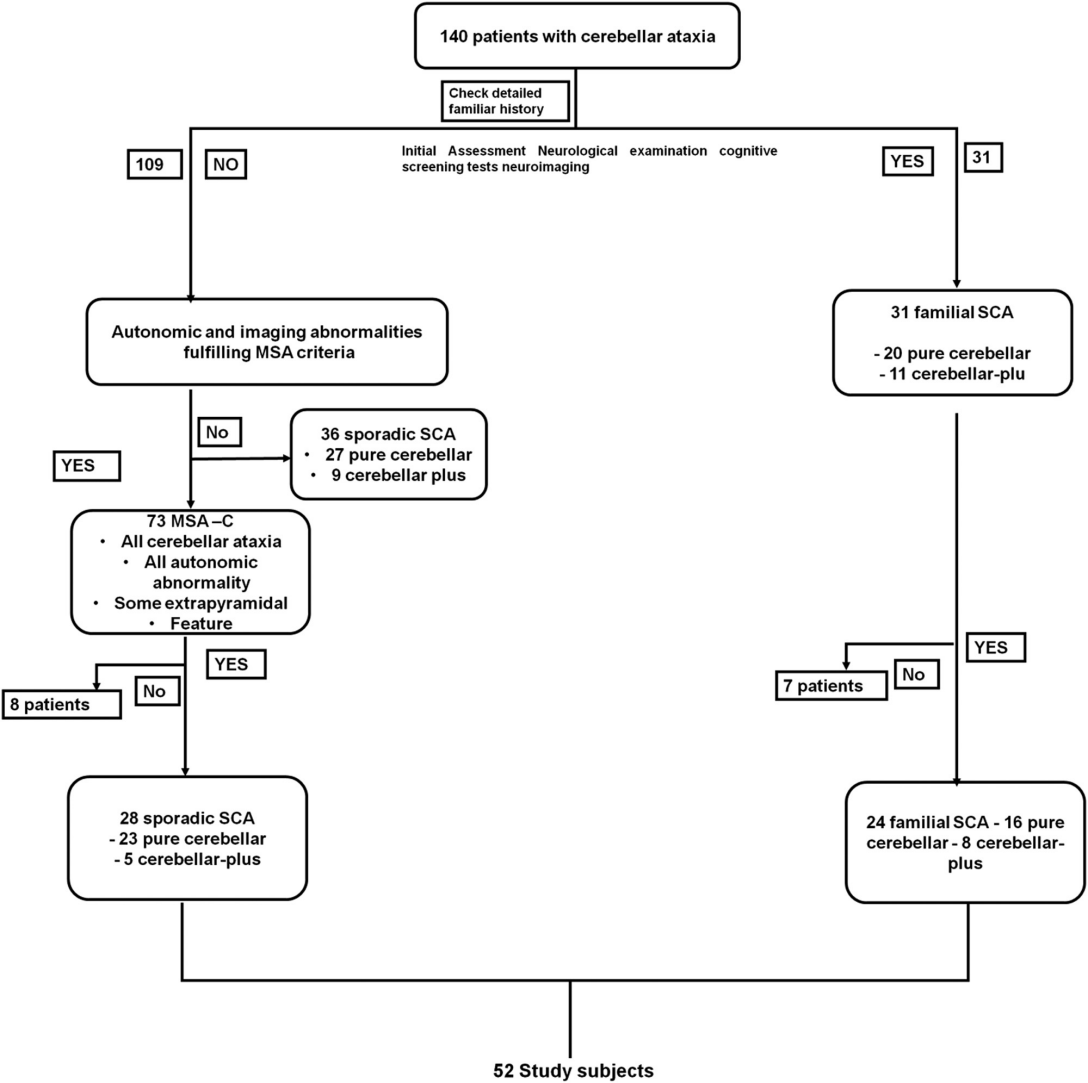
The process of patient recruiting.

One hundred forty individuals with CA remained after the initial assessment. Based on a thorough history encompassing 4 generations, 140 individuals with the condition were divided into 31 individuals with hereditary CA and 109 people with sporadic CA (Figure 1).

Only one patient with spontaneous CA had consanguinity (his parents are relatives). We eliminated 73 individuals with an MSA-C diagnosis using the established criteria. When sleep apnea/nocturnal stridor was indicated, we conducted head-up tilt tests, urodynamics accompanied by sphincter electromyography, and sleep studies. Of the remaining 67 individuals, 31 had familial CA, and 36 had sporadic non-MSA-C cerebellar ataxia. Twenty-eight individuals (80%) of the 36 people with sporadic non-MSA cerebellar ataxia asked for gene testing. Of the 31 individuals with familial CA, 24 (or 77%) asked to have their genes examined. The patients with familial CA served as the controls in the current investigation, including 52 individuals. Spearman’s rank correlation coefficient∼test and the Student’s *t*-test were used for the statistical study.

### 3.1 Inclusion criteria

All patients with CA referred to our neurology clinic throughout 8 years were the original inclusion criteria. Each patient was followed up for 5 years to determine the clinical diagnosis. The “Mini-Mental State Examination (0-30) and the Frontal Assessment Battery (0-18)” were used as cognitive screening tests, along with “MRI and single-photon emission computed tomography” used L-ethyl cysteine dimer, on all patients as part of the initial workup.

### 3.2 Exclusion criteria

Exclusion criteria included chronic CNS infections, hepatic encephalopathy, immune-mediated inflammation, vitamin deficiencies, Wernicke encephalopathy, regular alcohol use, diphenylhydantoin intoxication, superficial siderosis, and drug abuse, which were identified through a thorough nerve conduction study, examination, history taking, and blood tests.


**Informed consent:** Informed consent has been obtained from all individuals included in this study.
**Ethical approval:** The research related to human use has been complied with all the relevant national regulations, institutional policies, and in accordance with the tenets of the Helsinki Declaration and has been approved by the authors’ institutional review board or equivalent committee.

## 4 Results and discussion

### 4.1 Patient demographics

Imaging studies on 52 patients with CA revealed that 39% had cortical atrophy. Sixteen of these people had a genetic form of CA, whereas the remaining 23 cases were sporadic. According to the results of the neurological exams and tests, the patients had no signs of other CA.

The cerebellar hemisphere/vermis was the only part of the brain that showed any shrinkage or aberrant signals in any of the patients’ MRI studies. Thirteen individuals (sporadic 5, familial 8) had no history of CCA. Thirteen of the patients presented with various neurological disorders, including three with abnormal extraocular motion, two with extrapyramidal symptoms, six with pyramidal symptoms, three with peripheral neuropathy, one with myoclonus, one with epilepsy, and one with impaired vision. One patient had white matter lesions, and ten others had pontine atrophy, as seen on brain MRI images (Tables 1–4). Ten genes, SCA1, SCA2, SCA3, SCA6, SCA7, SCA8, SCA12, SCA17, SCA31, and dentatorubro-pallidoluysian atrophy (DRPLA), along with the CAG and TGGAA repeat length, were the focus of our gene analysis.

**Table 1:** Gene analysis results for sporadic cases

		Gene analysis result				
Patient	Sex	No. of repeat	Inheritance	Age (years)	Disease duration (years)	AAO (years)
S1	M	ND	Sporadic	39	31	8
S2	M	ND	Sporadic	55	10	45
S3	M	ND	Sporadic	59	6	53
S4	F	ND	Sporadic	63	1	62
S5	M	ND	Sporadic	66	4	62
S6	M	ND	Sporadic	66	18	48
S7	F	ND	Sporadic	68	5	63
S8	F	ND	Sporadic	69	5	64
S9	M	ND	Sporadic	75	23	52
S10	M	ND	Sporadic	75	13	62
S11	F	ND	Sporadic	76	2	74
S12	F	ND	Sporadic	80	1	79
S13	M	SCA1	Sporadic	65	55	10
S14	M	SCA1	Sporadic	77	2	75
S15	M	SCA2	Sporadic	56	10	46
S16	F	SCA3	Sporadic	71	5	66
S17	F	SCA6	Sporadic	58	14	44
S18	M	SCA6	Sporadic	65	18	47
S19	M	SCA6	Sporadic	65	25	41
S20	M	SCA6	Sporadic	65	22	43
S21	M	SCA6	Sporadic	69	15	54
S22	F	SCA6	Sporadic	72	22	50
S23	M	SCA6	Sporadic	73	17	56
S24	F	SCA6	Sporadic	73	8	65
S25	F	SCA6	Sporadic	75	6	69
S26	F	SCA6	Sporadic	80	16	66
S27	M	SCA8	Sporadic*	58	12	46
S28	F	DRPLA	Sporadic	16	16	0

Sporadic* in the notation is often used as a symbol to denote additional information or special circumstances.

**Table 2:** Clinical-imaging features of SCA for sporadic cases

	Neurological examination									Neuroimaging
Patient	AbNL EOM	Personality	Cbll ataxia	Epilepsy	Postural hypotension	Other	Cerebellar atrophy	Neuropathy	PA	Others
S1	−	−	+	−	−	−	+	−	−	−
S2	−	−	+	−	−	−	+	−	−	−
S3	−	−	+	−	−	−	+	−	−	−
S4	−	−	+	−	−	−	+	−	−	−
S5	−	−	+	−	−	−	+	−	−	−
S6	−	−	+	−	−	−	+	−	−	−
S7	−	−	+	−	−	−	+	−	−	−
S8	−	−	+	−	−	−	+	−	−	−
S9	−	−	+	−	−	−	+	−	−	−
S10	−	−	+	−	−	−	+	−	−	−
S11	−	−	+	−	−	−	+	−	−	−
S12	−	−	+	−	−	−	+	−	+	−
S13	−	−	+	−	−	−	+	+	+	−
S14	+	−	+	−	−	−	+	−	+	−
S15	+	−	+	−	−	−	+	+	+	−
S16	−	−	+	−	−	−	+	−	−	−
S17	−	−	+	−	−	−	+	−	−	−
S18	−	−	+	−	−	−	+	−	−	−
S19	−	−	+	−	−	−	+	−	−	−
S20	−	−	+	−	−	−	+	−	−	−
S21	−	−	+	−	−	−	+	−	−	−
S22	−	−	+	−	−	−	+	−	−	−
S23	−	−	+	−	−	−	+	−	−	−
S24	−	−	+	−	−	−	+	−	−	−
S25	−	−	+	−	−	−	+	−	−	−
S26	−	−	+	−	−	Vertigo		−	−	
S27	−				Loss of VS					
S28					Decreased					

“+” indicates presence of the feature. “−” indicates absence of the feature.

**Table 3:** Gene analysis results for familial cases

	Gene analysis result					
Patient	No. of repeat	Inheritance	Age (years)	Disease duration (years)	Sex	AAO (years)
F1	ND	AD	38	17	F	21
F2	ND	AD	59	11	M	48
F3	ND	AD	65	25	M	40
F4	ND	AD	76	7	M	69
F5	ND	AD	79	3	M	76
F6	ND	AD	79	7	M	72
F7	SCA2 37	AD	60	6	F	54
F8	SCA3 80	AD	32	14	F	18
F9	SCA3 71	AD	44	10	M	3,441
F10	SCA3 NA	AD	54	13	M	64
F11	SCA3 61	AD	70	620	M	51
F12	SCA3 64	AD	71	2	M	29
F13	SCA6 27	AD	31	13	F	36
F14	SCA6 NA	AD	49	20	M	41
F15	SCA6 24	AD	61	18	M	45
F16	SCA6 26	AD	63	19	F	60
F17	SCA6 22	AD	64	11	M	70
F18	SCA31 positive	AD	71	5	F	50
F19	SCA31 positive	AD	75	20	F	50
F20	SCA31 positive	AD	70	20	M	66
F21	SCA31 positive	AD	70	7	M	67
F22	SCA31 positive	AD	74	7	M	74
F23	SCA31 positive	AD	74	1	M	52
F24	SCA31 positive	AD	75	1	F	74

Sporadic* in the notation is often used as a symbol to denote additional information or special circumstances.

**Table 4:** Clinical-imaging features of SCA for familial cases

	Neurological examination									Neuroimaging
Patient	AbNL EOM	Personality	Neuropathy	Epilepsy	Cbll ataxia	Other	Postural hypotension	PA	Cerebellar atrophy	Others
F1	−	−	−	−	+	−	−	−	+	−
F2	−	−	−	−	+	−	−	−	+	−
F3	−	−	−	−	+	−	−	−	+	−
F4	−	−	−	−	+	−	−	−	+	−
F5	−	−	−	−	+	−	−	−	+	−
F6	−	−	−	−	+	−	−	−	+	−
F7	−	−	−	−	+	−	−	−	+	−
F8	−	−	−	−	+	−	−	−	+	−
F9	−	−	−	−	+	−	−	−	+	−
F10	−	−	−	−	+	−	−	−	+	−
F11	−	−	−	−	+	−	−	−	+	−
F12	−	−	−	−	+	−	−	+	+	−
F13	+	−	+	−	+	−	−	+	+	−
F14	+	−	−	−	+	−	−	+	+	−
F15	−	−	+	−	+	−	−	+	+	−
F16	−	−	−	−	+	−	−	−	+	−
F17	−	−	−	−	+	−	−	−	+	−
F18	−	−	−	−	+	−	−	−	+	−
F19	−	−	−	−	+	−	−	−	+	−
F20	−	−	−	−	+	−	−	−	+	−
F21	−	−	−	−	+	−	−	−	+	−
F22	−	−	−	−	+	−	−	−	+	−
F23	−	−	−	−	+	−	−	−	+	−
F24	−	−	−	−	+	−	−	−	+	−

“+” indicates presence of the feature. “−” indicates absence of the feature.

Of the 52 individuals with CA, 34 (65%) gene abnormalities were present. In the study, it was found that the most common type of SCA among the patients was SCA1 in 2 patients (3.8%), SCA2, followed by SCA3 in 6 patients (11.5%), SCA6 was observed in 17 patients (32.7%), SCA8 in 1 patient (1.9%), SCA31 in 5 patients (9.6%), and DRPLA in 1 patient (1.9%). No genetic abnormalities were found in the remaining cases. Gene abnormalities were identified in 11 out of 13 (85%) non-CCA cases when clinical-imaging subgroups broke down the results. Compared to CCA patients, where it was seen in just 23 of 39 (59%; *p* < 0.05), this is much higher. Patients with CCA had a greater average age of onset (55 years) compared to patients without CCA (40 years), while CCA patients had a shorter average duration of illness (12 years) compared to non-CCA patients (15 years).

Of the 28 people diagnosed with sporadic non-MSA-C cerebellar ataxia, 15 (56%) had underlying genetic disorders. There were 36.0% of instances involving SCA6 (10 individuals), 7.1% involving SCA1, 3.6% involving “SCA2, 3.6% involving SCA3, 3.6% involving SCA8, and 3.6%” involving DRPLA. There was no atypicality in the SCA31 gene. The remaining patients’ genomes had no anomalies found. Clinical imaging subtype analysis revealed that 5/5 (100%) of non-CCA patients had gene abnormalities. More than this was seen in CCA patients, which is to say 10/23 individuals (47.8%) with CCA had SCA6 anomalies; this was not statistically significant. Of the 24 individuals with familial CA, 18 (or 75%) had gene abnormalities. The most frequently mutated gene was SCA6, which was found in 8 patients (30%) and was “followed by SCA3 (4 patients, 20.9%), SCA31 (4 patients, 20.9 percent), SCA1 (1 patient, 3.6%), and SCA2 (1 patient, 3.6%).” In the remaining cases, there were no signs of gene abnormalities. There may be AD heredity in all familial CA cases.

Clinical imaging subtype analysis revealed that 5 out of 7 (71%) non-CCA patients had gene abnormalities. Similar results were shown in CCA patients, where 13/17 (76%) had abnormalities. If we examine age at onset and CAG repeat length (23 vs 25 years), one of them with SCA6 (17 patients), sporadic (10 patients) vs familial instances (7 patients), there was a greater age at onset (not statistically significant) and shorter CAG repeat length (23 vs 25 years) (Table 5).

**Table 5:** A contrast between sporadic and familial cases of SCA6

Patient	Age of onset (years)	Sex	Age (years)	Duration of disease (years)	Result of gene analysis (SCA6)	
					No. of repeat	Familiarity No (×) Yes (✓)
SP1	44	F	58	14	26	\[\times ]\]
SP2	47	M	65	18	23	\[\times ]\]
SP3	41	M	65	25	23	\[\times ]\]
SP4	43	M	65	22	NA	\[\times ]\]
SP5	54	M	69	15	26	\[\times ]\]
SP6	50	F	72	22	21	\[\times ]\]
SP7	56	M	73	17	21	\[\times ]\]
SP8	65	F	73	8	22	\[\times ]\]
SP9	69	F	75	6	22	\[\times ]\]
SP10	66	F	80	16	23	\[\times ]\]
F1	29	F	31	2	27	✓
F2	36	M	49	13	NA	✓
F3	41	M	61	20	24	✓
F4	45	F	63	18	26	✓
F5	45	M	64	19	NA	✓
F6	60	F	71	11	26	✓
F7	70	F	75	5	22	✓

## 5 Discussion

The most significant conclusions from this study are as follows: 57% of those with sporadic CA other than MSA-C have genetic abnormalities. Most prevalent were SCA6 gene anomalies (36%), then “SCA1 (7.1%), SCA2 (3.6%), SCA3 (3.6%), SCA8 (3.6%), and DRPLA (3.6%).” The SCA7, SCA12, and SCA31 genes were all normal. Thus, mutations in autosomal-dominant SCAs, especially SCA6, are not uncommon in sporadic CA [22,23]. “There were no statistically significant variations between sporadic and familial SCA6 cases in terms of maturity at onset (69 vs 59), disease duration (17 vs 13), or CAG repeat length (23 vs 25).” There were a total of 17 people with SCA6. This study is the first to examine ten genes for mutations associated with AD SCA in sporadic CA. Although the genetic component of sporadic CA has been underreported, we present evidence of a high-frequency rate (57%). Earlier studies only found a frequency of 2.8–18.8%. Thus, these new findings are striking. The gene testing method may have been different, albeit this cannot be confirmed. For instance, we examined ten genes to boost the likelihood of spotting genetic anomalies.

Furthermore, our individuals may have underreported their family dynamics. This cohort’s older time of commencement and varying penetrance of SCA6 anomalies may further account for the disease’s disproportionate prevalence [24]. This might be due to the families’ same cultural background or their tendency to live near one another [25]. In China, people with SCA6 are the norm. “Mutations in the gene responsible for SCA6 may be more common in sporadic CA since SCA6” appears to have the later clinical onset of all kinds of CA [26,27]. It was hypothesized that SCA6 is the most extraordinarily prevalent dominant SCA mutation in sporadic CA, and the current investigation verified this hypothesis.

For those affected by SCA6, a comparison between sporadic and familial instances reveals that the former has a later onset age (69 vs 59) and a shorter CAG repeat length (23 vs 25). As a result, sporadic instances of SCA6 may share less severe clinical-genetic characteristics with their relatives than do familial cases. But this must be sorted out with a more significant sample of patients. The idea that a novel mutation, mistaken paternity, incomplete penetrance, or the transmission’s demise of the parent before the start of clinical symptoms might all contribute to the development of SCA6 without a hereditary component seems consistent with these findings. Consistent with the results of the current investigation, it has been hypothesized that SCA6 and sporadic CCA share several clinical features. This is due to the localization of imaging findings and pathologic substrate in SCA6 to the cerebellar cortex.

## 6 Conclusion

In conclusion, sporadic CA is frequently caused by autosomal-dominant SCA mutations, notably SCA6. The precise cause is yet unknown. However, it is likely due to a greater age at the start and varied SCA6 penetrance. The amount of triplet repeats is absent from our study since some of our patients had their genes examined at other facilities. This may affect how the clinical-genetic connection turns out. Additional SCA genes, such as SCA11, were also inaccessible for analysis. The difference in the incidence of gene abnormalities between the current research and previous studies may be due to selection bias, including uncertainty about inclusion or exclusion and the lack of genetic history. Our study’s findings unambiguously show that autosomal-dominant SCA mutations, notably SCA6, are common in SCA despite the modest number of study participants. This discovery clarifies the need for appropriate patient care and the potential for future early treatment of this condition.
